# Unmasking legislative constraints: An institutional ethnography of linkage and engagement in HIV healthcare for African, Caribbean, and Black people in Ontario, Canada

**DOI:** 10.1371/journal.pgph.0000714

**Published:** 2022-09-21

**Authors:** Apondi J. Odhiambo, Lisa Forman, LaRon E. Nelson, Patricia O’Campo, Daniel Grace

**Affiliations:** 1 Dalla Lana School of Public Health, University of Toronto, Toronto, Ontario, Canada; 2 Yale School of Nursing, New Haven, Connecticut, United States of America; 3 St, Michael’s Hospital, Li Ka Shing Knowledge Institute, Toronto, Canada; PLOS: Public Library of Science, UNITED STATES

## Abstract

The Human Immunodeficiency Virus (HIV) epidemic significantly impacts African, Caribbean, and Black (ACB) immigrants in Canada. Health scholarship has revealed striking injustices within Canada’s public healthcare system that restrict access to healthcare and violate the human rights of ACB immigrants living with HIV who are marginalized. We conducted an institutional ethnography to comprehensively understand how HIV healthcare in Ontario is organized and experienced by ACB immigrants, focusing on unjust and discriminatory legislative frameworks and institutional practices regulating access to publicly funded healthcare resources and services. We interviewed 20 ACB immigrants and 15 healthcare workers, including specialists, primary care providers, immigration physicians, and social workers. We found a disjuncture between the organization of HIV healthcare in Ontario and how ACB immigrants experienced access to care. We uncovered how immigration, public health and healthcare laws and related institutional practices intersect to produce structural violence which create barriers and missed opportunities to timely linkage and engagement in HIV healthcare. Black immigrants’ accounts revealed that they underwent mandatory HIV under the Immigration Medical Examination policy (IME) without providing informed consent and receiving pre and post-test counselling. Furthermore, Black immigrants did not receive referrals and were not adequately linked to care following HIV diagnosis. Troubling encounters with immigration and public health state agents and healthcare legislative barriers including difficulty finding a physician, the 3-month waiting period under the Ontario Health Insurance Plan (OHIP), long wait times, lack of drug coverage, and stigma, discrimination, and anti-Black racism shaped and affected Black people’s linkage and engagement in HIV care. We elucidate how the legislative and structural organization of healthcare regulated and constrained health service access for ACB immigrants living with HIV, including their ability to “achieve” HIV undetectability.

## Introduction

Advances in healthcare have transformed HIV infections from a fatal disease into a chronically manageable condition [1, 2]. Recognition of antiretroviral therapy’s (ART) preventative and health benefits informed the development of the HIV healthcare cascade or care continuum [3]. The cascade consists of stepwise stages of healthcare that a person progresses through from HIV testing, diagnosis, linkage and engagement in medical care, and initiation of ART to achieve viral suppression and an undetectable viral load [4]. Each progressive step of the cascade is concerned with the ability of a person diagnosed with HIV to be linked and remain actively engaged in healthcare. “Linkage to care” is a critical step in the cascade that refers to a person’s ability to successfully enter the healthcare system and complete a first medical clinical visit after an HIV diagnosis. Gaining linkage to care is a precursor to the initiation of ART and a pathway to viral suppression. Early linkage of people who receive a positive diagnosis to care, initiation of ART, and continued engagement in medical care improves health outcomes and optimizes the overall wellbeing of people living with HIV by suppressing the amount of the virus in the blood to undetectable levels. Viral suppression enhances the quality of life of people living with HIV by improving physical functioning, reducing infections, lowering mortality, and effectively eliminating the risks of ongoing HIV transmission [2]. Research shows that delays in linkage to medical care after HIV diagnosis lead to faster disease progression, poor health outcomes, and mortality [5].

Global public health scientists, healthcare professionals and policymakers in Canada and globally have positioned biomedical approaches as the “magic bullet” to ending the HIV epidemic [6, 7]. Currently, the focus is on getting all people diagnosed with HIV linked and engaged in the healthcare cascade. In 2016, Ontario’s government launched the provincial strategy called *HIV/AIDs strategy 2016–2026*: *Focusing Our Efforts*: *Changing the Course of the HIV Prevention*, *Engagement and Care Cascade in Ontario*. This strategy, which centers the cascade, aims to explain how the healthcare system works to successfully link, engage, and retain people living with HIV in healthcare and achieve and maintain an undetectable viral load [8]. In 2014, the Joint United Nations Programme on HIV/AIDS (UNAIDS) launched the 90-90-90 Fast-Track initiative, which aimed to get 90% of all people with HIV to know their status, 90% of those diagnosed receive antiretroviral treatment, and 90% of those on treatment achieve viral suppression by 2020 [9]. To end HIV as a global public health threat by 2030, the “90” targets were expanded and replaced by the “95-95-95” 2025 targets. The United Nations (UN) Member States including Canada committed to achieving the 95–95–95 targets by ensuring that all people living with HIV have access to medicines, treatment, and diagnostics by 2025 [10].

Despite recent scientific advancements and global efforts and initiatives, HIV/AIDS epidemic continue to wreak havoc nationally and globally. Studies show that marginalized and racialized communities, particularly African, Caribbean, and Black (ACB) people are being left behind in HIV response [11, 12]. Black people, who comprise 3.5% of Canada’s total population, represent a diverse community of persons who are Canadian born and immigrants who have been in Canada for decades or recently immigrated from African and Caribbean countries [13]. In this study, the terms “Black” and “ACB” people or immigrants are used interchangeably to represent racial identity, ethnocultural diversity, origin, and history of immigration and slavery. Black people remain disproportionately impacted by HIV in Ontario, Canada. In Ontario, they represent 25% of newly reported HIV cases while constituting only 5% of the provincial population [14, 15]. Black people living with HIV continually experience challenges accessing healthcare along the continuum of care, including timely linkage and engagement [16]. We argue that existing biomedical approaches have not been the hoped-for “magic bullet” to ending HIV among ACB people living with HIV, primarily because of unjust laws, policies, institutional practices, and racialized ideologies that produce inequities and violate the right to health.

### Laws, inequities and right to health

Canada ratified and committed to several international human rights treaties which recognize, promote, and protect the right to health. Article 12(1) of the International Covenant on Economic, Social and Cultural Rights (ICESCR) recognizes “the right of everyone to the enjoyment of the highest attainable standard of physical and mental health” [17, 18]. The General Comment No. 14 issued by the UN Committee on Economic, Social, and Cultural Rights (CESCR) that explains article 12.1 on the right to health stipulates in paragraph 19 that governments have obligations to “ensure health insurance and services and healthcare facilities are accessible to all, especially the most vulnerable of marginalized sections of the population, in law and in fact, without discrimination on any of the prohibited grounds” [19]. The general comment 14 further imposes on states an obligation “to respect” the right to health and refrain from interfering with the enjoyment of this right either by restricting access to health services and enforcing discriminatory policies and institutional practices. Despite being a signatory of the ICESCR, many aspects of Canada’s legal systems, policy, institutional practices, and political ideologies are harmful and contribute towards the violation of the right to health for certain individuals and groups based on race, socio-economic status and immigration class. ACB people living with HIV in Canada. Scholars and human rights advocates acknowledge that ACB people living with HIV experience inequities that hinder their ability to navigate the complex Canadian healthcare system, access HIV healthcare and treatment, achieve optimal health, improve their quality of life, and realize their right to health [20, 21].

The Canada Health Act (CHA) is the federal legislation regulating Canada’s publicly funded healthcare system. The CHA works to “ensure that all eligible residents of Canadian provinces and territories have reasonable access to “medically necessary” hospital and physician services on a prepaid basis, without charges related to the provision of insured health services” [22]. Canada’s healthcare system, also known as Medicare, illuminates a social contract between the Canadian federal, provincial and territorial governments, medical practitioners, and the public grounded on CHA’s principles of universal, transferable, comprehensive, accessible, and public healthcare [23]. The Interim Federal Health Program (IFHP) is a special federal fund that regulates healthcare access for refugees and offers urgent and necessary care to refugee claimants who have successfully applied for permanent legal status in Canada and are awaiting adjudication and determination of their case. Canada’s provincial and territorial governments each have independent health insurance programs with considerable variations in the level of public coverage for prescription medications [24].

Notwithstanding CHA’s principles and fundamental values of fairness and equity informing the healthcare system and its commitments to realizing human rights, Canada still fosters inequities that constrain access and people’s right to health and healthcare. Although CHA ensures universal coverage of “medically necessary” physician and hospital care, no province provides universal and comprehensive healthcare coverage to its residents [25]. This is because the CHA does not define “medically necessarily” healthcare services. As such, there is no legal mandate for provinces to provide outpatient medication coverage for all residents. This legislative gap presents barriers to drug coverage in Canada for certain groups of people. Canadian residents with public provincial health insurance plans do not have access to comprehensive and universal healthcare coverage for medically necessary services and prescription drugs including outpatient prescription and over the counter medications [26, 27]. A limited set of public programs provide drug coverage to a selected subset of Ontarians: those who are 65 or older, those on social assistance, whose prescription drug costs are high compared to their annual incomes, or those with disabilities requiring income support [28–31]. In contrast, Manitoba and British Columbia do not provide drug coverage for residents above 65 years old. Empirical studies show that people living with HIV encounter difficulty accessing ART due to a lack of drug coverage and oppressive social assistance policies [32–34]. The three-month waiting period imposed on new immigrants under OHIP also delays access to healthcare services [35]. New immigrants to Canada experience difficulty obtaining immediate care due to long wait times before making the first contact with a physician and accessing healthcare services [36].

### Structural violence

In this study, we argue that Canada’s unjust social structures including laws, policies, institutional practices, and racialized ideologies produce and perpetuate structural violence, leading to the insufficient impact of biomedical approaches to HIV prevention, healthcare, and treatment. Structural violence is defined as- “the violence of injustice and inequity” which is “embedded in ubiquitous social structures [and] normalized by stable institutions and regular experiences” [37]. Structures refer to the taken-for-granted and institutionalized social relations and arrangement such as legal, political, economic and ideologies including patriarchy, slavery colonialism, neoliberalism, poverty, and discrimination based on race, immigration status and class that shape how individuals and group interact with and within social systems [37].

John Galtung coined the term structural violence to conceptualize hidden or invisible forms of violence embedded within social relation that cause harm and suffering [38]. Galtung argues that structural violence “shows up as unequal power and consequently as unequal life chances” [39]. Thus, structural violence is a “way of describing social arrangements that put individuals and populations in harm’s way. The arrangements are structural because they are embedded in the political and economic organization of our social world; they are violent because they cause injury to people” [40]. Structural violence which is produced and implemented through “tools of oppression” entrenched within bureaucratic systems create inequities resulting into unequal opportunities and life chances [38]. Paul Farmer describes structural violence as harm and suffering exerted systematically and indirectly by unjust and oppressive socio-political systems and manifesting through restrictive policies and bureaucratic institutional practices that limit access to services. Furthermore, the impacts of structural violence on health are compounded and perpetuated by restrictions on the right to health, including lack of access to adequate and quality healthcare for those who are structurally disadvantaged and oppressed [41, 42]. Akhil Gupta asserts that structural violence is enacted through the everyday practices of bureaucracies and one, therefore, needs to look closely at those everyday practices in order to understand why violence coexists within care and why, paradoxically, it is often found in practices of welfare” [43].

This background clearly demonstrates that social structures organizing and coordinating healthcare delivery in Canada structurally marginalize certain individuals and groups and restrict the right to health including healthcare. However, the ways in which these social structures produce and perpetuate inequities and social injustices that constrain ACB people’s individual agency and capacity to access HIV healthcare and treatment remain relatively understudied.

HIV response community have overemphasized individual risk factors and biomedical discourses and neglected the role of structural determinants such as laws, policies, racialized ideologies, political and economic interests, racism and institutional interests in driving HIV vulnerabilities and transmission risks and shaping health outcome [1, 2, 6]. The biomedical “magic-bullet” approaches are devoid of societal and structural factors that produce and perpetuate structural violence, and influence health outcome of [44]. Global public health scientists and health policy advocates warn that a narrow focus on piecemeal solutions including biomedical technology, patient compliance and the basic science interventions limited to HIV “obscures other aspects of the epidemic, such as its intersection with structural violence, thereby making meaningful and effective interventions (aimed at ending HIV) difficult to establish” [44, 45]. It is essential for HIV response to extend beyond biomedical and behavioural approaches and integrate determinants of health, the “circumstances in which people grow, live, work and age” and which drive inequities -the unfair and avoidable differences in health status between individuals and among groups [46]. Amon advocates for “structural rights”, which include “a right to know about the barriers that impact one’s care, a right to access to treatment after one’s status have been known, and a right to protection under the law …. against discrimination, as well as a right to confidentiality” [44, 47]. Under the right “to know” and “to be known”, social realities which are often regarded as unreliable and ignored produce evidence that unmask broken and failing systems [44, 47]. Therefore, the intricate social realities of ACB people living with HIV, a “target population” of authoritative and institutional knowledge about HIV, and the health workers who are mandated to implement knowledge calls for an analytic framework that “allow people-centered evidence to add up, to travel, and to matter publicly and comparatively” [44].

This study is a part of a larger institutional ethnographic project that used the concept of structural violence to problematize the social organization of HIV healthcare and treatment from the perspective of ACB people living with HIV in Canada [34]. The inquiry is concerned with the institutional framing and implementation of HIV healthcare and treatment practices, and how it happens that well-intending biomedical interventions to address HIV/AIDS epidemic fail to generate expected health outcome for ACB people living with HIV. Therefore, this study empirically questioned assumptions entrenched in the dominant biomedical knowledge about HIV response, showing not only how local experiences are shaped by powerful social structures, but also how social structures undermine biomedical HIV response practices and efforts to end HIV locally and globally. We conceived this project as a response to disproportionate impact of HIV and biomedical HIV response approaches for ACB people living with HIV, largely attributed to social structures shaping material conditions [20, 21]. We argue that biomedical, ideological, and “institutional” knowledge about HIV prevention, healthcare and treatment are “ideas and social forms of consciousness [which] originate outside the experiences [for instance of ACB people], a forced set of categories into which we must stuff the awkward and resistant actualities of our worlds” [48]. Efforts to prevent, manage and end HIV/AIDS epidemic are devoid of ACB people’s experiences and needs, leaving them behind in HIV response. A particular concern is the inequities and injustices arising from their implementation within a social context with unjust laws and policies, and racialized ideologies.

We applied institutional ethnography analytic approaches to navigate different forms of knowledge about HIV healthcare and treatment and uncover how ACB people’s efforts of accessing HIV healthcare and treatment are constrained by the taken-for-granted discursive processes and textual practices emerging from unjust laws and policies regulating access to health resources in Canada. The study empirically examined discursive connections between everyday experiences of ACB people living with HIV, social context, institutional practices, and the global architectural discourses shaping HIV prevention, healthcare, and treatment. The aim was to identify and make visible how unwittingly ACB people and their actualities are systematically excluded from research and HIV healthcare and treatment discourses and practices. The analytic objective was to create a detailed map of the work of accessing HIV healthcare and treatment, starting from the standpoint and experiences of ACB people living with HIV. Expert knowledge from lived experiences were traced back to the realm of the social organization of HIV healthcare and treatment, explicating empirically the discursive processes that link the local, national, and global biomedical discourses, and laws, policies and institutional practices that regulate access health resources. Explicating these complex processes put this study into the context of global public health in relation to HIV response. It raises several important questions about the role of unjust social structures in derailing global HIV response efforts. While biomedical approaches are being celebrated globally for transforming HIV response, serious critiques also need to be made of the invisible structural forms of violence that social structures produce and perpetuate through inequities and social injustices, particularly for structurally marginalized populations.

Important to note, this analysis in no way disregards the significant contributions biomedical approaches and global instruments are making in global HIV response. Instead, we argue that unjust and inequitable social structures constrain access to biomedical interventions inherently causing harm and suffering to ACB people living with HIV. Therefore, this analysis calls for critical refection on the role of social structures in HIV response and seeks strategies to work from within them integrating lived realities and needs of structurally marginalized populations. Such analysis provides a critical perspective which may ignite renewed advocacy and contribute positively towards policy reforms and program interventions.

## Method

### Institutional ethnography

Institutional ethnography is a critical social science and method of inquiry that makes a “radical departure” from mainstream sociology, ethnography, and other qualitative approaches that follow conventional approaches “in which people [are] the objects […], whose behaviour [is] to be explained” [49, 50]. Smith critiques that such ideological knowledge which are embedded in power relations represent only the perspective of those in power.

Dorothy Smith describes institutional ethnography as an “alternative sociology” for people. The analytical focus of institutional ethnography are “social relations”, which are the often taken-for-granted and invisible “extended relations that coordinate multiple settings translocally” and are located in “how people’s activities or practices are coordinated” [49]. According to Smith’s theory of “social organization of knowledge”, ruling relations create forms of knowledge that structure how members of a society view themselves and the social world in which they live [48]. Therefore, institutional ethnography examines how people’s experiences are organized by ruling relations, paying attention to socially organized systems of knowledge. Institutional ethnographers are also interested in explicating how people’s lives are governed both within and beyond the local context by text-mediated ruling relations. Texts are documents that individual activities activate and bring to life such as laws, policies, and professional guidelines [51].

An institutional ethnographic inquiry focuses on understanding the “work” that people do by analyzing what they say and what they know about their work as expert knowers and doers. Work in institutional ethnography refers to anything that people do with intent and requires time, effort, and skill [49]. McCoy, an institutional ethnographer, states that the healthcare of a person living with HIV involves a complex, daily work process that loops from the home and everyday spaces of the individual into the sites of professional medical services and back again [52]. It also involves the work done by healthcare providers engaged in clinical and social HIV healthcare [53]. This study expands the concept of “healthwork” foregrounded by Mykhalovskiy and McCoy that analytically focuses on how institutional relations within and beyond healthcare systems organize the work that people do to maintain their health [54]. The ‘healthwork’ processes under scrutiny in this study is the work devoted by ACB people living with HIV to care for their health, including navigating the Canadian Healthcare system to access HIV healthcare. Work is, therefore, the experiential pathway to understanding the social organization of HIV healthcare and how ACB people experience healthcare.

Empirically, institutional ethnography inquiry begins from a ‘research problematic,’ defined as a guiding perspective that “directs attention to possible research questions…that have yet to be posed or of puzzles that are not yet formulated as such, but are ‘latent’ in the actualities of our experienced worlds” [48]. As such, research problematic becomes an entry point to the study of a social organization of knowledge through experiences of certain individuals that concerns a researcher [49]. Methodologically, entering a study from a particular research problematic requires creating knowledge of how ruling relations are organized and operate from the standpoints of individuals interacting with those very relations. Theoretically, the premise is that ruling relations are instituted by people who have differing experiences and knowledge because they are positioned distinctively in relation to the systems. Smith refers to the distinctive position as the”standpoint” [49]. From a social justice stance, entering the research from the standpoint of structurally marginalized individuals or groups ensures that people’s lived realities provide the basis for and entry point to understanding how ruling relations produce and perpetuate structural violence through inequities and social injustices. The aim is to learn from people’s “experience and with tracing how their everyday lives and doings are caught up in social relations and organization concerting the doings of others, although they are not discoverable from within the local experience of anyone” [49].

As a method of inquiry, institutional ethnography aims “to link local experiences to broader social and global processes, which are not always immediately apparent at the local level” and mostly invisible to us [55]. Therefore, the analytic goal of institutional ethnography is to “produce for people what might be called ‘maps’ of the ruling relations and specifically the institutional complexes in which they participate in whatever fashion” [49]. Empirical mapping help institutional ethnographers to explicate “how [certain things] are put together,” “how things are going on” [56], how people interact with ruling relations which govern their social world, and how institutional practices are organized in ways that create barriers for those in the “standpoint location” [50, 57]. The work of mapping the often invisible social relations and how they organize and shape “the actualities of people’s everyday lives and experiences” allows the ethnographer to take sides and work in the interests of the standpoint group [49].

Institutional ethnography has a longstanding history in HIV and Acquired Immunodeficiency Syndrome (AIDS) research scholarship. Researchers have used Institutional ethnography to problematize social relations that organize people’s lives at the local level, starting from people’s everyday standpoints and experiences. In the early 1980s, George Smith blended institutional ethnography and activism to investigate the everyday work that people living with HIV in Canada, whose social world was organized by discriminatory ideologies such as “disease vectors” or “AIDS as a fatal disease,” did to access healthcare services including ART in order to survive [58]. Smith’s research elucidates that the inequities people living with HIV continue to experience result from injustices deeply rooted in the Canadian healthcare system, legislative frameworks, and institutional practices for decades. Several institutional ethnography studies have since explored the institutional coordination of access to ART and other social services for people living with HIV [54, 59].

#### Establishing the research problematic and entering the research

Since immersing herself in public health research, the first author has been interested in providing a contextual understanding of how ACB people interact with Canada’s healthcare system while seeking healthcare. The first author’s lived experiences as a Black and African immigrant, early-career scholar, and activist working and doing research with ACB communities informed her interest in this line of inquiry. During community engagement activities, the author heard ACB people living with HIV sharing disturbing experiential accounts trying to access HIV healthcare and treatment. The literature was full of authoritative knowledge about HIV prevention, healthcare, treatment, and related concepts such as “linkage”, “engagement”, “retention”, “adherence” and “undetectable viral load”. It was difficult to see how this authoritative knowledge were relating to the everyday realities of ACB people living with HIV-especially those structurally disadvantaged and experiencing various forms of precarities. There were tensions between how authoritative biomedical and institutional knowledge about HIV prevention, healthcare and treatment were envisaged to work and implemented, and the subjective experiential accounts of ACB people encountering inequities in health determinants [20, 21, 23]. Through our research, literature review and activism work, we learnt that contextual and structural factors such as laws, policies and institutional practices organizing and governing systems and social world of people restrict right to health, including access to essential health and social services for certain groups of people, particularly for racialized communities and immigrant groups experiencing various forms of precarity. It is in these tensions that our research is situated and informs our research problematic.

### Sampling and recruitment

The first author purposively sampled and recruited 20 ACB people living with HIV through healthcare sites and community-based AIDS organizations (ASOs) (n = 20). The participants were recruited if they self-identified as HIV positive, as African, the Caribbean and Black, were at least 18 years of age and lived in Toronto. Purposive sampling techniques were also used to recruit 15 healthcare providers and health policy actors involved in delivering HIV healthcare (n = 15). The recruitment and interviewing process was iterative. As we learnt about who was involved in providing healthcare to ACB people, we identified the next person to contact and what issues to discuss.

### Data collection

Institutional ethnographers conceptualize alternatives forms of knowledge starting from people’s everyday local experiences mostly through in-depth interviews, participant observations, text analysis and researcher’s reflections of their everyday life before investigating how their experiences are coordinated translocally with specific institutional processes and social relations [60]. In this inquiry, we took the standpoint of ACB people living with HIV who had experienced challenges interacting with systems and institutional processes of HIV healthcare and treatment, and whose lived realities are often ignored, taken for granted and obscured by abstract authoritative knowledge and public legislation and policy. While the everyday work and activities of service users (such as ACB people) and health workers are organized and shaped by multiple and intersecting systems of knowledge, they are often not involved in the conceptualization and production of related concepts and intervention or healthcare.

We used in-depth interviews and textual analysis as primary data collection methods. Interviews provided an opportunity for standpoint informants (ACB people) and health workers to share their experiences and highlight how ruling relations coordinate their activities in relation to HIV healthcare and treatment. Biehl and Petryna note that people experiencing a particular problem should not be perceived not just as victims but also as “agents of health…who can help us to better understand the larger systems and policies in which lives actually unfold” [44]. They emphasize that “by bringing the voices of those living and dying with HIV/AIDS to bear on the legal and structural analyses of policy, critical studies of global health not only rescue voices left behind by other epistemologies but also offer a way to uncover the inadequacies of current approaches and to orient the development of new ones” [44]. Therefore, there is validity in centering the of perspectives of those who are systematically excluded from research, and this value outweighs the reliability problems associated with self-reporting through interviews.

Before the interviews, the first author asked these participants to complete a brief demographic questionnaire. We used an interview guide to explore ACB people knowledge and perspectives about HIV healthcare and treatment. The questioning was done in a way the first author could begin to trace ACB people knowledge and activities related to HIV healthcare and treatment connected with those of health workers. ACB people were prompted to describe the work they do to access HIV healthcare with questions such as: How did you go about accessing care? How did you get connected with an HIV specialist or family doctor, made clinical appointments and visits, engaged in laboratory work, accessed prescriptions and drugs? Who did you interact with during HIV healthcare? What materials and orders were you given? What challenges did you encounter and how did they shape your experiences accessing care?

To build a strong empirical investigation beyond the experiences of ACB people, we conducted interviews with people located at different sites within the institutional system of healthcare including health workers such as nurses, social workers, pharmacists, physicians, clinicians, and health policy actors. A separate interview guide was used to explore health workers’ activities of delivering HIV healthcare along with their insights on health system barriers and systemic factors shaping ACB people’s engagement in HIV care. The guide included questions about their work, their challenges, and how to improve linkage and healthcare engagement. Throughout the interview process, the first author developed an analytic practice of listening for and asking about texts that regulated HIV healthcare work while conducting interviews with ACB people and healthcare providers. Health workers accounts of their work processes revealed their positions within health systems and HIV healthcare and treatment related practices.

### Ethics and consent

The study was approved by the University of Toronto Research Ethics Board. We had initially planned to conduct all the interviews through face-to-face. Towards the end of data collection processes, we received ethics approval to conduct the rest of the interviews by telephone or through zoom due to COVID-19 pandemic and related guidelines. All participants who participated in face-to-face interviews provided written consent at enrolment. Participants who engaged in the interviews virtually provided written consent via email or verbally through telephone or zoom. All verbal consents were recorded, dated, and signed by the principal investigator. All interviews were audio-recorded and transcribed based on participant consent.

### Analytic and mapping process

Drawing on fieldwork carried between June 2019 and May 2020 in Toronto, we explicated the lived experiences of ACB people living with HIV and empirically elucidated the connections between their everyday lived realities and the extra-local ruling relations that govern them, in contrast to abstract globalized dominant biomedical knowledge systems that socially organize HIV response. All interviews were audio-recorded and transcribed. The research process was iterative as the first author went back and forth between data collection and analysis of interview transcripts and texts. This process involved reading all transcripts multiple times, locating various actors’ work practices within the HIV care system, and tracing their connections to other external actors’ work. To ensure a rigorous critical inquiry, the first author asked the following reflexive questions throughout the research process: What are the everyday experiences and daily HIV healthcare activities of ACB people? Are there any activities coordinated at the local level? What are the texts and institutional processes coordinating sets of activities at the local level? How might this coordination of activities be connected to the Canadian healthcare regime or other extra local institutions? The informative and illuminating standpoints of ACB people living with HIV provided the departure point from which the first author identified and mapped dominant textually mediated processes.

Using institutional ethnography analytics of mapping, we moved outward to examine the social organization and implementation of HIV healthcare services within the Canadian health system [48, 49, 61]. The first author identified health work processes across participants’ accounts of their experiences accessing HIV healthcare and treatment. Indexing was used as an analytic tool to group together instances of healthwork process across participants’ accounts [56]. For example, every time a participant mentioned something that had intent and took time and effort (i.e., gaining linkage into HIV healthcare), an index was created (i.e., “healthwork of linkage into HIV healthcare). The particulars of each healthwork process were then grouped using NVIVO.

We used the exertion of structural violence as an analytic lens to map out the text-mediated processes emerging from the everyday HIV healthcare work experiences of ACB people and their healthcare providers, and the institutional practices that go into healthcare delivery [49, 51]. The analytic end-goal was to map and understand how regulatory forces, institutional practices, and related structural factors enter people’s local settings to constrain the work of linkage and engagement in the HIV healthcare cascade. Therefore, we explicated the disjuncture between the HIV healthcare cascade’s ideological organization and the actual experiences of ACB people across the cascade. We then tracked and unmasked the broader social structures shaping and governing HIV healthcare and treatment. More specifically, we mapped and described the ways laws, policies, and institutional and professional processes intersect to shape how HIV healthcare and treatment is organized and experienced by ACB people within Canada’s public healthcare system. Mapping the cascade spectrum unmasked how inequities and barriers resulting from the intersection of immigration, public health, and healthcare legislative frameworks and institutional practices constrain the work of linkage and engagement in care for Black people. The mapping process also identified workaround practices ACB people used to navigate barriers as they sought linkage and engagement in care. The map revealed how ACB people’s experiences and healthcare providers’ work practices were connected and coordinated by known or unknown social and ruling relations.

## Results

### Participant demographics

Among the ACB participants living with HIV, 60% of the participants were African (n = 12) and 40% Caribbean (n = 8), with 55% identifying as women (n = 11) and 45% men (n = 9). Most participants identified as heterosexual (80%). The sample also included individuals that self-identified as gay and queer (20%). Participants ranged in age from 25 to 67 years old. Black participants self-identified as immigrants who had immigrated to Canada. Over one-third had been in Canada for less than one year (35%, n = 7), 40% less than five years (n = 8), and 15% between 5–20 years (n = 5). Participants reported having precarious immigration status with 30% having non-status (n = 6), 40% refugee claimants (n = 8), 10% asylum seekers (n = 2), 10% international students (n = 2) and 10% and permanent residents experiencing precarity (n = 2). Over half of the participants had healthcare coverage (55%, n = 11), while 45% had none (n = 9). All participants in this study had no drug coverage, with over 50% relying on social assistance to access drug benefits. All participants reported an annual income of less than $20,000, with experiences of homelessness and housing instability. The health workers included healthcare providers (n = 10), panel physicians (n = 2), and frontline healthcare workers (n = 3) providing HIV healthcare to ACB communities.

### Mapping the social organization of HIV healthcare and treatment for ACB people who are “medically insured”

**Fig 1:**
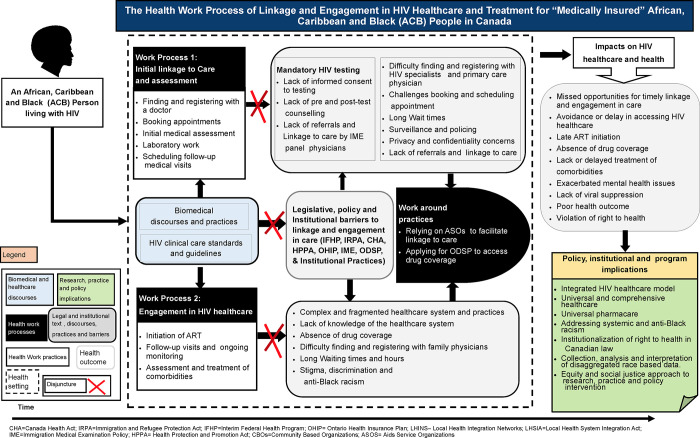
The health work of linkage and engagement in HIV healthcare and treatment for African, Caribbean, and Black people living with HIV in Canada.

This article maps findings from the experiences of eleven “medically insured” ACB people living with HIV (n = 11). In this study, “medically insured” refer to people who had access to public health insurance coverage, such as IFHP and OHIP at the time of the study. The main finding of this article is that ACB people with healthcare coverage require timely linkage and consistent engagement in HIV healthcare and treatment. However, social, and ruling relations organizing and governing healthcare delivery in Canada produced inequities and social injustices that constrained medically insured ACB people’s efforts of accessing HIV healthcare and treatment, subjecting them to various forms of structural violence. Fig 1 summarises ruling relations and textually medicated processes that shaped ACB people’s lives and created barriers to HIV healthcare and treatment access within Canada’s healthcare system, and the healthwork processes that they engaged in to navigate these barriers, and gain linkage and engage in HIV healthcare and treatment. In the section below, we provide an extensive description of how HIV healthcare and treatment discourses and ruling relations shaped access and delivery of HIV healthcare and treatment from the perspective of ACB people and healthcare workers.

#### The work of initial linkage to care and clinical assessment

The ACB people living with HIV and healthcare providers commonly expressed how a HIV positive diagnosis triggered a need to be linked to care and access HIV healthcare. Participants’ experiential accounts revealed the critical role medical doctors play in initiating linkage to HIV healthcare. The Ontario Clinical Care Guidelines for Adults and Adolescents Living with HIV in Ontario, which uses a “patient journey” approach, indicates that initial linkage to care is the “patient’s first contact encounter with the clinical team” that “sets the stage for sustained care” [62]. Therefore, a patient is said to have linkage to care where there is a “confirmed [first] care visit with intake and complete initial assessment by a nurse, nurse practitioner or physician.”

The ACB immigrants identified the following immigration and healthcare legislative and institutional practices as presenting barriers and missed opportunities for timely initial linkage to care and assessment: a) mandatory HIV testing practices under IME policy, including the absence of consent and counselling, lack of referrals and connection to care by IME panel physicians, and troubling practices and encounters with immigration and public health state agents; and b) difficulty finding an HIV specialist, booking appointments, and long wait times.

*Injustices under the IME policy*. All the ACB immigrants we interviewed reported that they went through mandatory IME after applying for Canada’s permanent legal status. Doctors working in hospitals and panel physicians affirmed that the immigration law mandates all immigrants who claim refugee status at the port of entry or inland to undergo a mandatory “medical checkup.” A textual analysis of the immigration legislation reveals that medical programming and prospective immigrants’ health is legislated by the Immigration and Refugee Protection Act (IRPA), S.C.2001, c.27, s. 18 [63]. The IRPA and supporting regulations set out the medical requirements for people who apply to stay in Canada permanently. Section 16 (2) (b) of IRPA requires that all foreign nationals aged 15 years and above who request to remain in Canada permanently must undergo an IME to determine whether they have a “*disease*, *disorder*, *disability or other health impairment that might reasonably be expected to cause excessive demand health or social services*” [64]. The IRPA provides that all IMEs are conducted by panel physicians. Panel physicians are licensed medical doctors designated by Immigration, Refugees and Citizenship Canada (IRCC) to conduct IME.

*Mandatory HIV testing without informed consent and counselling*. ACB participants experienced that they experienced double injustice under the IME policy. First, they noted that they were subjected to mandatory HIV testing. Secondly, Secondly, they explained that they were not informed and consulted about the HIV test, they did not provide informed consent, and the potential consequences of the test were not explained either through pre or post counselling. ACB immigrants who did not know their HIV status when claiming refugee status explained that they got diagnosed during the “refugee checkup [IME process].” It also did not occur to many immigrants that they were vulnerable to or living with HIV. The immigrants described being in a state of shock, disbelief, confusion, fear, and denial when panel physicians informed them of their HIV positive status. ACB immigrants who knew their HIV positive status were equally shocked to learn they were tested for HIV during the IME. Participants expressed concerns with the panel physicians’ attitude, noting that the physicians lacked empathy and were disconnected from their feelings while informing them about the HIV diagnosis. A Black African woman in her 50s who was a newcomer to Canada and residing in a shelter explained the injustices she encountered under IME policy:

*I was in the shelter*. *I remember being a newcomer in Canada*. *They called me from the shelter and told me*, *“We need to see you*,*” and we set an appointment*. *I went not knowing why they needed to see me*. *When I went*, *the first thing they said was*, *“Nancy*, *we have checked your blood and we have found two things*. *You have a lot of sugar in your urine*, *have you ever been diabetic*?*” I said no*, *but I do not know*. *The second thing they said*, *“Nancy*, *we want to let you know that we have found out that you are HIV*. *Have you ever been checked in Africa or what happened*?*” I said*, *yes*, *I checked two times*, *but maybe it was not in the system [body] because they did not find it*. *I was a little bit shocked*. *I am still in denial*, *like what happened*.

Textual analysis revealed that since 2002, IRCC conducts mandatory routine HIV screening on all applicants 15 years of age and older who request and apply for Canadian permanent residency, including refugees and asylum seekers, temporary residents, and those under 15 years who have certain risk factors such as having received blood or blood products or having a known HIV-positive mother. According to IME policy, HIV screening must occur in sanctioned Canadian and foreign medical offices under a responsible panel physician [65].

The IRCC developed the “Canadian Panel Member Guide to Immigration Medical Examinations” as a handbook for licensed doctors who serve as panel physicians and conduct IME [66]. The guide structures the process of mandatory HIV testing and instructs responsible panel physicians to obtain the applicant’s consent and declaration and provide pre and post-test counselling to applicants who test HIV positive. The guide stipulates that panel physicians must have applicants who test HIV positive sign the Acknowledgments of HIV “post-test counselling form” (IMM 5728 form) and report confirmatory HIV testing to immigration and public health officers [67]. The applicants must sign the IRCC Client’s “consent and declaration form” as an affirmation that they have consented to the “collection and release of IME information related to the administration of Canada’s IRPA or to the protection of the health and safety of Canadians.” Contrary to the guidelines, none of the participants we interviewed recalled ever providing verbal or written informed consent or receiving pre-test and post-test counselling before and after HIV testing. A woman in her mid 40s who underwent IME together with her husband and underage son explained that they received their HIV diagnosis through email:

*They [immigration clinic] actually sent a mail to my husband…The mail they sent was to tell us about our status*, *my son’s status*, *his own status and every other thing*, *our general body condition*. *And there and then we discovered that [Positive HIV stats]*, *It [email] didn’t show if I’m still undetectable or my son is still undetectable*.

*Missed opportunities for referrals and linkage to care*. ACB immigrants’ accounts demonstrated that mandatory IME practices presented missed opportunities for linkage to care. Immigrants reported that the absence of informed consent and counselling coupled with the fear of HIV diagnosis, denial, limited information about HIV, and the uncertainty of how to access care had detrimental effects on their ability to seek and gain timely linkage to care. Panel physicians did not make referrals or provide them with adequate information on accessing HIV care or managing their diagnosis. They noted that they lacked knowledge about how to “navigate the healthcare system” and access HIV care. A Black man in his mid 30s who was diagnosed with HIV through the IME explained the lack of adequate referrals and linkage to care by IME panel physicians:

*I did not know what to do because nobody told me anything*. *They [immigration doctor] just gave me an address and said*, *“go to this place*, *go to that place*.*” Navigating that*, *getting around without resources and getting to know where to go was quite a challenge for me*.

Panel physicians determined that immigrants’ “lack of knowledge” of Canada’s healthcare system was a major barrier to accessing HIV healthcare and treatment. While IME physicians felt that they had the moral duty to link applicants who tested positive to care, they were legislatively limited by the directives outlined in the panel physicians’ “handbook” and under IRPA. The IRPA states that panel physicians are “third-party physicians” whose duties and responsibilities are different from “treating physicians” and limited to medical services related to the IME.

*Troubling practices and encounters with immigration and public health agency state agents*. Most ACB immigrants felt that panel physicians were more concerned with reporting their HIV-positive diagnosis to the public health agency than assisting them in navigating the system and getting linked to care. Literature shows that discourses and practices of new public management (NPM) principles that rely on text-mediated forms and frameworks have reorganized government institutions, separating them into “single purpose organizations” to increase responsibility and accountability pressures [68, 69]. The effects of new public management within health institutions are reflected in the Health Protection and Promotion Act, 1990 (HPPA), RSO, 1990, c. H.7 and participants narratives. Section 25 of the HIPPA provides licensed physicians with the duty to report certain clinical conditions to public health or law enforcement authorities as part of their commitment to protecting the public from harm. It states:

*Physicians must report to the Medical Officer of Health of the health unit in which the professional services were provided when*, *in the course of providing professional services*, *they have formed the opinion that an individual has or may have a disease of public health significance and is not a patient in or an out-patient of a hospital*.

The above extract demonstrates that HIPPA regulates panel physicians and public health officers while granting them autonomy to pursue public health goals, as narrated by Black participants.

All participants who underwent mandatory testing explained that panel physicians informed them that they would be “reported” to “public health” if they tested HIV positive. Participants described receiving “random calls” from the public health agency after undergoing mandatory HIV testing. These participants were concerned about their privacy and confidentiality during their encounters with public health agents. Public health staff coerced participants into discussing their sexual health and HIV positive status over the phone without considering whether they could talk openly or not; forcing ACB immigrants to accept public health calls while in public settings such as at work, the mall, or among friends. There was no opportunity to express whether they were comfortable talking or needed to reschedule the call. A Black man in his mid 40s who had received a call from public health agent while in a public setting narrated:

*“I had a call from the public health*. *They said “Matin [pseudonym]*, *you are HIV*. *That one even came to me as a shock because*, *I was in the mall*, *and they are calling you and they are not even counseling you*. *They just tell you “Matin we know that you are HIV and whatever…” He went directly to the point*. *And then quickly he is telling me and it’s over the phone*. *He doesn’t know if it is me or not*. *He is telling me all that information in like 3*, *5 minutes*. *There is no preparation*, *there is no sensitivity around my status*. *So that I felt as a person who works in public health myself*, *as a person who has been able to help and support people in disclosure*, *I felt that was quite unethical*.

An ACB man in his late 30s who was a refugee and living in a shelter when we interviewed him complained that public health staff breached his privacy when they called the shelter and left information that made the shelter staff suspicious of his health. Shelter staff questioned why public health was looking for him and whether he had an infectious disease. An ACB participant in her early 50s recalled how she was informed about her HIV-positive status over the phone by a public health staff: *“We have received your immigration results and they show you are HIV positive*. *You know you are supposed to start HIV treatment*. *Go see the doctor and get medication and you have to ensure you don’t infect anyone*.”

Traumatizing, coercive, discriminatory, stigmatizing, and punitive first encounters and experiences with immigration and public health agents negatively shaped immigrants’ attitude and perceptions about seeking HIV healthcare. The knowledge that they would be policed and surveilled made the immigrants fearful and confused. Some immigrants noted that fear of being considered a burden to Canada’s healthcare system and the risk of their application for legal immigration status being rejected and deported made them avoid or delay seeking HIV healthcare. These interactions also triggered immigrants’ mental health issues, including depression and anxiety. Another participant, an ACB man in his mid 40s contested that public health surveillance practices were oppressive, unethical, and created privacy concerns, leading to avoidance of accessing HIV healthcare:

*You cannot pass such an important decision over the phone*. *That right there can damage someone for the rest of their lives*. *The first person you are in contact with determines how you access services in this country*. *It can make someone just run and hide because they threaten to report*. *They use words like ‘surveillance*.*’ Where I am coming from*, *those are harsh words*. *It’s like somebody will be following you and knowing what is going on or happening*. *Who knows*, *how are they going to use this information*? *What will happen to me*?

ACB participants generally felt that the public health staff were more concerned about surveilling and policing their sexual practices than ensuring they were linked and engaged in HIV healthcare. The public health staff did not provide resources and information about how they could go about getting an HIV specialist and access HIV healthcare.

*Difficulty finding a doctor*, *booking appointments and long wait times*. ACB immigrants and healthcare providers indicated that difficulty finding and registering with an HIV specialist, booking appointments, and long wait times were barriers to accessing care. HIV specialty and primary-care clinics told ACB immigrants seeking linkage to care that the physicians were not taking on new patients. Several doctors reported that sometimes they could not take on new patients because their “practice [was] full.” Specialists who are also “clinical scientists” explained that they could only allocate a limited amount of time to clinical work. One HIV specialist expressed: *“I’m supposed to only spend 20% of my time doing clinical work and I cannot see all my patients in that time*. *I’m already doing more than I should because I love doing clinical work and seeing patients*.”

ACB immigrants noted that some doctors refused to take them on as new clients because they had *“the big brown document from immigration [refugee protection claimant document]*.*”* Refugee protection claimant documents prove that a patient is eligible for coverage under the IFHP. Doctors noted that the administrative processes of determining refugee claimants’ eligibility for IFHP, and billing are “complex” and “bureaucratic.” Doctors explained that it is their responsibility to confirm an individual’s eligibility for IFHP before providing any healthcare services.

They noted that refugee claimants seeking care must therefore show their refugee protection claimant document. Those doctors interested in providing services to IFHP beneficiaries must first register with the Medavie Blue Cross insurance company and comply with all their terms and conditions for billing and reimbursement purpose [70]. Doctors who were registered as IFHP providers at the time of the study reported that the requirement to obtain preapproval from the insurance company before providing certain service sometimes delayed access to HIV healthcare and treatment. They explained that the approval process is often burdensome and time-consuming, with wait times of up to three months. Healthcare providers noted that these institutional complexities sometimes contribute to some physicians’ unwillingness to take on refugee claimants as new patients.

ACB participants who were eligible for OHIP but had recently moved to Ontario from out of province or as new immigrants explained that they had to wait for three months before accessing healthcare. Healthcare providers described how they turn patients away because of the three months wait rule. One physician explained:

*It takes three months for the OHIP to get activated*. *I have stories about pregnant women from Ghana*. *The husband sponsored her when he got permanent residence*. *They are both HIV positive*. *She came*, *and she was pregnant with HIV*. *They knew about the three-month rule*. *They could not access care for three months*, *waiting without OHIP coverage*. *She wasn’t on antiretroviral therapy*. *So that is a barrier*, *that three-month OHIP period waiting*.

ACB immigrants commonly noted that all these challenges resulted in long wait times before they gained linkage and access to care.

*Workaround practices to linkage to care*. Immigrants identified community-based organizations (CBOs), social workers, and AIDS service organizations (ASOs) as the primary conduits to linkage in HIV healthcare. Several immigrants reported that they had to register with local organizations and disclose their HIV status to social workers before getting referrals and linked to care. Immigrants had to work very hard to get connected to local organizations and get support. Grace explained: *“I had friends who were working in the HIV sector who I had worked with back in Africa…So they were linking me*.*”* Philip reported: *“I had a friend who was a member of the clinic and church who helped me get connected*.*”* Several immigrants expressed concerns that they had to disclose their HIV status to many people before receiving any support. Some immigrants reported delaying seeking support upon realizing that they had to first “disclose” that they were living with HIV. HIV doctors explained that they received several referrals from local ASOs and community healthcare centers (CHCs) with whom they have developed close partnerships and collaboration.

#### The work of engagement in HIV healthcare

The accounts of ACB participants and healthcare providers and textual analysis of the Ontario HIV clinical care guidelines described the work of engaging in HIV healthcare as involving initiation of ART, a series of follow-up visits after diagnosis and initial linkage and assessment, and ongoing monitoring and care [62]. Healthcare providers elaborated that they “*come up with treatment plans*” together with patients whose results show readiness and are willing to start ART. Ongoing monitoring and care involve finding and scheduling appointments with extended healthcare systems including laboratories, pharmacies, primary healthcare physicians, and support services. Physicians expressed that they recommend testing viral loads one month after initiating treatment to ensure the virus’s suppression. If a patient is stable, physicians recommend checking a patient’s viral load every six months to ensure that the viral load remains undetectable. During clinical visits, physicians issue laboratory requisitions and drug prescriptions to their patients. Physicians elaborated that they check to ensure patients are not developing toxicity from their medication during follow-up assessment

ACB people and healthcare providers identified and described the following as HIV healthcare engagement barriers: (a) complex and fragmented healthcare system and a lack of knowledge of the system; (b) absence of drug coverage and difficulty accessing HIV treatment; and (c) difficulty finding a family physician and long wait times scheduling an appointment to manage comorbidities.

*The complex healthcare system and structural barriers*. Fragmented institutional practices and ACB people’s lack of knowledge of how the healthcare system operates were barriers to effective engagement in HIV healthcare. ACB participants commonly found “navigating” the complicated institutional processes to engage in HIV care activities challenging. An ACB man in his 40s expressed that there was no “*one-stop-shop where patients can access all services*.*”* A physician explained that immigrants seeking HIV healthcare and treatment “*walk around blindly because they do not know the system*. *These immigrants come from another country to a new country with a different system*. *Ontario also has its system of healthcare delivery*.” The complex organization of Canada’s healthcare system, coupled with a lack of knowledge of the system, made it difficult for the immigrants to access HIV care and services promptly.

*Absence of drug coverage and the work of applying for social welfare assistance*. Participants also identified the absence of drug coverage as a significant barrier to HIV treatment access. They described the different workaround practices they engage in to navigate the drug coverage barrier and access to HIV treatment. In Ontario, the OHIP does not provide prescription drug coverage [29]. When prescribing medication, physicians must establish whether their patients have drug coverage or not. One physician elaborated that before giving a patient a prescription, he would ask questions like, “*Can you fill it*? *Where are you going to fill it*?” This physician further elaborated that: “*for immigrants who need medication and have absolutely no coverage*, *one has to figure out what they are going to do to access their HIV medication*, *[…] which could be really challenging*. *We have to scramble around to somehow get some way to get them on treatment*.” For patients with no coverage, physicians must assess whether they need to apply for Ontario Disability Support Program (ODSP), are eligible for Trillium Drug Program (TDP), or have no access at all [31]. The TDP is a drug program that offers financial assistance for Ontario residents who have high prescription drug costs compared to their net household income [71]. The TDP provides coverage for specific prescription drugs. Physicians treating ACB immigrants explained that TDP is only accessible to individuals who meet specific eligibility criteria. One physician explained: “*People cannot fill in the Trillium forms unless they have filed their taxes for one financial year*.*”* The immigrants we interviewed were not eligible for TDP because they were unemployed and had not yet filed their taxes. The publicly funded Ontario Drug Benefit (ODB) program provides prescription drug coverage for Ontarians with a valid OHIP card who are aged 65 or older, on social assistance, or registered for the TDP [31]. However, the ODB only covers people that are 65 years and over. A physician noted that *“people who fall through the crack [HIV healthcare and treatment cascade] are those between 25 years and 65 years*, *majority of whom are HIV positive immigrants without drug coverage*.*”*

The ODSP is a provincial social assistance program that helps people with disabilities who need income support to access health benefits such as prescription drug coverage under the Ontario Disability Support Program Act [28]. All ACB immigrants interviewed noted that they rely on Ontario social assistance programs to access HIV treatment. Healthcare providers reported that they advise their patients without private insurance to apply for the “ODSP.” Successful ODSP applicants received a drug card, which they used to access HIV treatment. The work of applying for ODSP is a perfect example of what Smith calls institutional categories that must be filled for an individual to fit institutional procedures [49]. To qualify for ODSP, the applicant must meet both financial and medical disability criteria as provided for under the ODSP Act. HIV is considered a disability under section 49(1) of the ODSP Act, which defines disability as:

*A person can have a disability if (a) the person has a substantial physical or mental impairment that is continuous or recurrent and expected to last one year or more; (b) the direct and cumulative effect of the impairment on the person’s ability to attend to his or her personal care*, *function in the community and function in a workplace*, *results in a substantial restriction in one or more of these activities of daily living; and (c) the impairment and its likely duration and the restriction in the person’s activities of daily living have been verified by a person with the prescribed qualifications*. *(Ontario*, *1997*, *c*. *25*, *Sched*. *B*, *s*. *4(1)*.

The assessment must be documented in a specific way to ascertain that the applicant is entitled to receive the service.

*Difficulty finding doctors*, *long wait times*, *and delays in accessing healthcare services*. Black people accessing HIV healthcare are screened for comorbidities or co-infections such as diabetes, bone density issues, liver and kidney issues, and high blood pressure as part of HIV healthcare. People who accessed HIV care through specialty clinics were advised by their specialists to find a family doctor who would be responsible for providing them with primary care and managing comorbidities. Black people were frustrated by how difficult it was to find a family physician to commence treatment for their comorbidities, leading to suboptimal health. They faced challenges in booking appointments and experienced long wait times before seeing a family physician. It was also challenging finding a family doctor who was knowledgeable about HIV and was taking new HIV patients. On ACB participant, a woman in her 30 expressed: *“I have an ear infection and it has been challenging me for almost a month now*, *I couldn’t get an appointment with my general practitioner [family doctor]*. *They could only get it two months later”*. Another ACB participant, a male in his 50s explained: “*it took two months to get an appointment for my stomach because there were a lot of patients before me*. *My HIV specialist wrote a letter to them*, *and they put me on their waiting list*.*”*

*Experiences of stigma*, *discrimination*, *and anti-Black racism*. The intersections of stigma, discrimination and anti-Black racism based on race, HIV status, and origin shaped immigrants’ healthcare access experiences. These factors determined what type of healthcare services ACB people could access, how they received these services, and how healthcare provided treated them. ACB people expressed that they experienced stigmatizing and discriminatory practices and attitudes from healthcare providers, including long waiting hours and substandard services because of their race, HIV status, and place of origin. An ACB male participant in his 40s, who accessed care through emergency department visits, felt his privacy and confidentiality were breached when a nurse inquired about his HIV status before other people and failed to attend to him immediately. Another male participant in his 20s, who indicated that he “waited for five hours” before seeing a doctor, felt that a non-Black nurse who refused to attend to him and instead looked for a Black nurse did so because of his Blackness and HIV status. Black participants also felt that they are subjected to mandatory screenings of infectious diseases while seeking HIV care because of the perception that Black people from Africa and the Caribbean are “disease carriers.” A Black frontline health worker based at a local ASO who self-identified as HIV positive expressed:

*There are times that you feel race is used as a label to identify or direct what type of healthcare is given to you or your clients*? *I think when the doctors look [screen]*, *they look for TB*. *There is an assumption [that] everyone from Africa has TB*. *If you go to the hospital coughing*, *you will be quarantined because you are from Africa*. *It had nothing to do with your TB*. *When you are asked about where you have been*, *they expect you to have Ebola or something because you are coming from Africa*. *When it comes to HIV itself*, *there is an assumption that Africans are the ones that bring HIV*, *so the way you get treated is almost like*, *“these diseases carriers*.*” So*, *race plays a part in how you receive that care and how fairly you are treated*. *Someone else who is Caucasian or born here is not treated the same way you are being treated*.

Several ACB immigrants elaborated that healthcare and surveillance practices drive anti-Black racism, stigma, and discrimination, create fear of the healthcare system and influence HIV healthcare services engagement.

#### “Instrumental” health work of supporting ACB people navigate structural barriers

As described in previous sections, ACB people encounter structural barriers and challenges that constrain their ability to access HIV healthcare and treatment and achieve and sustain undetectable HIV viral load. Health workers expressed that many ACB people living with HIV are new immigrants with lack of knowledge of the Canadian culture and how to navigate the complex healthcare system, encounter language barriers and lack social network. Further, ACB people encountered significant challenges associated with lack of access to social determinants of health including housing, food, and health coverage. Health workers detailed that they support ACB people to navigate structural barriers to access health resources including HIV healthcare and treatment. We refer to this effort as “Instrumental health work.” Health workers explained that they conduct social assessment to determine the individual needs of each patient. A social worker based at a HIV clinic defined instrumental work, stating:

*My job as a social worker*. *is one that I will refer to as instrumental*, *which means anytime I am helping someone achieve something*, *I have to fill lots of piece of paper*. *It’s the application for housing*, *it is the application for social assistance*, *it is helping them get resources from different places*, *whether it is legal education or healthcare in the community or anything like that*. *So*, *I do a lot of instrumental work with people*, *specifically*, *when I first meet them on their first visit because that is when I do the first assessment and I find what is missing in their lives*, *that would be helpful*. *A lot of those things they are instrumental things*.

Healthcare providers explained that they engage in instrumental health work because it is important to protect people’s human rights including right to health. A health provider explained:

*I want to make sure that people are protected under the laws*, *and they have right to health*, *healthcare*, *and right to citizenship*. *All these things are important because they [ACB people] are people and they live here [Canada] now*. *So*, *they should have same right as everyone else and unless they are assisted*, *they won’t know exactly how to do that*. *There is a necessity that people need to live somewhere*. *And that is what I am there to help them through*, *provide for them a decent standard of living*.

Health workers described the kind of instrumental health work they do to support ACB people living with HIV including resourcing for basic healthcare needs and social determinants of health. A health provider explained:

*The first time they [patients] come to the clinic*, *I will meet with them*. *And because I have been doing this for a while*, *I know questions to ask them*. *So*, *the needs I am helping them meet would be housing…*, *where they are getting income if they are getting any income whatsoever*, *and then I help them figure out how to apply for Ontario Disability Support Program*. *Access to medication can be a big one sometimes for people depending on their immigration status*. *Sometimes it is helping people resource just basic healthcare coverage*: *doctor visits*, *having blood taken*, *laboratory work and access to medication*, *making sure that they have ongoing medication as you know HIV is 100% medication driven*. *A big factor it is making sure people secure medication*.

Black participants and healthcare workers reported that they engage in the healthwork process of applying for ODSP to mitigate barriers associated with lack of drug coverage. Black participants indicated that their doctors and social workers based in ASOs, and shelters helped them get into ODSP. Healthcare workers explained that they advise patients without insurance and unable to afford treatment out of pocket to apply for ODSP. An ACB female participant in her 40s explained how she became a recipient of ODSP: “*I got in ODSP because my family doctor was kind of moving me towards going on ODSP*. *I was not really pressed about going on ODSP and then I realized that this was a way for me to get my medication covered*.” Black participants detailed the healthwork of applying for ODSP, which includes the textual work processes of sourcing and filling of forms. Healthcare providers play a critical role in ODSP application processes. Black people explained that when applying for ODSP, they are given forms that they must fill together with their HIV doctors to determine if they meet the criteria of a person living with a disability. An ACB male participant his 30s stated: “*They [ODSP] give you a form to take to your doctor and your doctor must fill it*. *The doctor must write you come here [clinic] for this and this [nature of disability]*.” Another ACB participant, a woman in her 40s who was working at the time of the interview but “did not have coverage [drug coverage],” explained:

*I had to prove that I could not afford to buy medication with the income that I was getting*. *So*, *I had to get my doctor’s prescription*, *go to a pharmacy*, *get a quotation about how much my medication costs in a year*, *and then submit it to the ODSP worker before starting my medication*. *I also had to report my income every month*.

The work process of applying for ODSP was seen as objectifying applicants and oppressive. Black people considered the process of applying for ODSP as complicated, tiresome, characterized by long waiting times, and standardized. Strict eligibility requirements and long waiting times made it difficult for Black people to qualify for ODSP and access timely HIV treatment. Black people without comprehensive health coverage reported that they avoided seeking healthcare and treatment for HIV and comorbidities because of their inability to pay for prescription medication. Participants noted that their peers informed them that their refugee claims and applications for the permanent residence could be rejected because of their dependence on social assistance and being a burden to Canadian health and social systems. These participants avoided applying for social assistance to access drug coverage and HIV treatment, fearing that being on the social welfare database would jeopardize their application for permanent legal status and put them at risk of deportation.

Health workers described how they assist ACB people to access HIV medication through compassionate drug programs provided by pharmaceutical companies. However, compassionate drug programs are not sustainable. One healthcare provider explained: “*We often look to the pharmaceutical companies to see if they would give compassionate supply [medication]…That is a terrible system because it is only in the generosity of a company what if they decide to stop?*”

Healthcare workers commonly acknowledged that ACB immigrants experience institutional barriers to timely linkage to HIV healthcare. They suggested that an integrated care model or what they commonly referred to as a “circle of care” could enable easy and quick access to panel physicians, family doctors, specialists, and social workers from community organizations. Health workers conceptualized circle of care (CCM) model as a framework that integrates formal and informal care networks and aims to improve HIV healthcare for ACB people living with HIV. The CCM takes a person-centered approach where those making decisions and providing formal and informal care are linked to the person seeking care [72, 73]. One HIV specialist expressed that *“it is ideal to be connected to a family doctor*, *an ASOs or CBO*, *and HIV specialist…Kind of a like a triangle…We are all working together to service that individual in a shared decision-making model*.*”*

## Discussion

This institutional ethnography illuminates a disturbing disjuncture between biomedical discourses and practices of linkage and engagement in care across the cascade and undetectability, which is essential for effective management of HIV and achieving of optimal health, and how Canada’s healthcare system and institutional practices are socially organized. Study findings elucidate how structural violence embedded and hidden within Canadian immigration, public health, and healthcare legislative frameworks and institutional practices produce inequities that constrain Black people’s healthwork of gaining timely linkage and engagement in care, contributing to the violation of their right to health. Suboptimal mandatory HIV testing processes absent of informed consent and counselling, oppressive public health surveillance practice, healthcare system barriers including difficulty finding a doctor and scheduling appointments, long wait time and fragmented healthcare services, and stigma, discrimination, and anti-Black racism are forms of structural violence that constrain linkage and engagement in HIV healthcare. Moreover, punitive immigration and public health policies structurally violate Black people’s human rights through discriminatory practices of surveillance and policing.

Under the Immigration and Refugee Protection Act, all immigrants applying for legal permanent status in Canada must undergo IME [63]. ACB participants experienced multiples forms of injustices and structural violence under the IME policy. First, ACB participants were subjected to mandatory HIV testing. Secondly, panel physicians failed to follow HIV testing and counselling guidelines outlined in IME policy and clinical guidelines outlined in several documents in and beyond the Canadian context [66, 74–76]. Black immigrants subjected to mandatory HIV testing under IME policy were not educated about HIV, did not provide informed consent, and did not receive pre-and post-test counselling. News of an HIV diagnosis was a distressing and life-altering event for Black immigrants characterized by fear of the diagnosis, denial, and uncertain consequences of living with HIV. However, panel physicians disregarded the mental and emotional state of the immigrants by not providing post-test counselling. The HIV/AIDS Legal Network emphasizes that “inadequate counselling is not only unethical and poor practice; it is contrary to the legal doctrine that medical interventions require a patient’s informed consent” [77]. HIV clinical care guidelines recommend that an individual newly diagnosed with HIV should see a knowledgeable healthcare provider within two weeks following diagnosis for clinical assessment, initiation of ART, and onward clinical care [78].

Studies show that HIV testing is a critical starting point in thinking about linking people who test positive to healthcare and treatment [79]. Quality pre and post-test counselling further help facilitate linkage to care and treatment initiation [62, 76]. However, immigration panel physicians did not make referrals or link immigrants to care. Additionally, immigrants’ lack of knowledge of the Canadian healthcare system made it challenging for them to gain timely linkage to care. Immigrants were left to struggle with the disturbing reality of HIV diagnosis and the challenge of navigating a complex and fragmented healthcare system to access HIV care. Findings show that reinforcing public health practices of surveillance and policing override newly diagnosed Black immigrants’ healthcare need of timely linkage and engagement in care. Panel physicians prioritized notifying public health agencies about HIV diagnosis over linking immigrants to care, as public health policy requires panel physicians and diagnostic centers to forward all HIV positive results to the responsible public health agency. These findings are consistent with those from other studies that have examined the impact of immigration and public health policies and institutional practices on HIV testing, linkage to healthcare, psychosocial support, and engagement in the HIV care cascade [65, 80–82].

With the evolving landscape of biomedical practices, including the universal test and treat and scaling up of ART, HIV testing and counselling gaps during IME and public health practices are hugely concerning [83]. Panel physicians and public health agents are the initial points of contact and provide an opportunity to facilitate timely linkage to care for immigrant applicants who test HIV-positive during the IME. The disregard of HIV testing guidelines, and prioritization of public health surveillance practices over Black immigrants’ mental health after a distressing diagnosis or making referrals constitute structural violence. Therefore, the unjust and suboptimal mandatory HIV testing practices and prioritization of oppressive public health practices present barriers and missed opportunities for timely linkage and engagement in care, initiation of ART, and attainment of optimal health outcomes for ACB immigrant communities [84]. These oppressive, threatening, violent, discriminatory, and traumatizing immigration and public health practices, and troubling encounters with state agents, negatively influenced Black immigrants’ ability and attitudes to seeking HIV healthcare services.

Black immigrants were also concerned about the breach of privacy and confidentiality by both immigration and public health professionals. Uncertainty about living with HIV, fear of surveillance, policing, non-voluntary disclosure, criminalization or deportation, mental health issues, and mistrust of the healthcare system and professionals resulting from these unjust practices contributed to the delay and avoidance of accessing HIV healthcare. The institutionalization and normalization of structural violence through immigration and public health surveillance practices lead to increased suffering as the bodies of Black immigrants diagnosed with HIV are pathologized and HIV stigma, discrimination, and anti-Black racism are perpetuated by compounding the idea that people from African and Caribbean are disease carriers and danger to public safety. Further, institutionalization of structural violence also materializes when the responsibility of navigating the complex and bureaucratic healthcare system and structural barriers and negotiating access to care is left to the individuals rather than taken up as a collective issue. These results extend Bisaillon’s work on “immigration medicine” and other studies that have looked at mandatory HIV testing and its implications on human rights and healthcare access [82]. In line with basic screening standards, it is indispensable for all HIV testing programmes to have a structured process that ensures newly-diagnosed individuals gain linkage to HIV healthcare services and treatment [85].

Despite accessibility to public health insurance plans, problematic and bureaucratic policies and institutional practices organizing healthcare delivery in Canada constrain ACB immigrants’ efforts to gain timely linkage and engagement in HIV healthcare [27]. ACB immigrants experience long wait times before getting linked to care and accessing HIV healthcare service. Complex and fragmented healthcare delivery models, lack of knowledge of how the system is organized and operated, and bureaucratic institutional practices make it challenging for ACB immigrants to find and register with HIV specialists and primary care providers, and book medical appointments. Specialty clinics and primary care settings offering HIV healthcare service experience increased service demand because of shortages of physicians with HIV knowledge and doctors’ competing interests and responsibilities between clinical practice and research [86]. Study results further revealed that most physicians and clinics are often unwilling to take on new HIV-positive clients, denying them access to care. ACB immigrants also experience long waiting times for various laboratory and diagnostic services. Long wait times and delays in healthcare access are consequences of the “fee-for-service” (FFS) model [87]. In Canada, healthcare is delivered by independent doctors, clinics, or a network of hospital physicians who operate through the FFS business models. Under the FFS model, physicians generate business revenue by scheduling and billing one issue per visit [23]. ACB immigrants who are new residents to Ontario are denied access to healthcare services due to the three-month waiting period policy under OHIP, forcing individuals to delay seeking linkage to HIV care or discontinue HIV treatment. Studies have associated the three-month waiting period with late diagnosis of comorbidities, worsening disease progression, and poor health outcomes [35].

While refugee claimants in our study were eligible for healthcare services through IFHP, bureaucratic institutional processes related to determining eligibility and billing produce long wait times and barriers to accessing HIV healthcare. Finding physicians and specialists accepting new patients on IFHP is often challenging for ACB immigrants. Bureaucratic institutional practices associated with IFHP and the discriminatory and racist discourses about refugee claimants make healthcare facilities such as walk-in clinics, hospitals, and doctors’ offices problematize and ostracize refugees with IFHP. Complex and burdensome administrative and billing procedures, lack of knowledge of how IFHP works, and what medical services are insured under IFHP disincentive physicians from accepting IFHP [88, 89]. The confusion in the application for IFHP and the long-lasting consequences on healthcare access for IFHP beneficiaries resulted from policy changes introduced to the IFHP between 2012 and 2016 by the federal government. These confusions strengthen barriers to accessing healthcare services by asylum seekers and refugees as they are denied care regularly [90, 91]. A report produced by Wellesley Institute and results from several studies have highlighted the continued impact of IFHP policy changes on immigrants’ healthcare access [88, 92, 93].

Cumulatively, ACB immigrants reported two to three months waiting time from diagnosis, finding referrals, and registering with an HIV specialist or primary care physician clinical team for consultation and clinical assessment to getting treatment. Several studies conducted across Canada have found similar findings [94–96]. Our study findings are also consistent with other global studies that report long wait times in healthcare systems in the United Kingdom and United States [97, 98]. Studies show that long wait times for medically necessary services have detrimental consequences such as delays in diagnosis and treatment of comorbidities, deterioration in health conditions, poor health outcomes, exacerbated mental health and economic sufferings [96, 99]. As a result of these systemic barriers, ACB immigrants heavily relied on ASOs and local community organizations to find HIV specialists and family doctors. Healthcare providers and clinical HIV care guidelines championed an integrated healthcare delivery system as an innovative model for improving service delivery, reducing long wait times, hours at clinics [62]. However, the high level of autonomy, competing interests, and the FFS incentive discouraged physicians from being part of an integrated healthcare delivery system. Studies show that the FFS model’s revenue incentive makes physicians address one medical issue per visit and rush through their clinical appointments to serve more patients per day, leading to accumulated health problems and more medical visits [100].

Although Black people in this study were`medically insured under OHIP and IFHP, they lacked drug coverage. The Ontario province covers prescription drugs through a patchwork of public insurance coverage that is not equitably accessible to all its residents [30]. Individuals who have legal status in Canada and have been residents of Ontario for three months can apply for the TDP, which requires annual payments of deductibles depending on household income [101]. However, in this study, ACB people were unemployed or over-represented in precarious and low-wage employments, and were therefore ineligible for private drug coverage, could not afford medication out-of-pocket, and were illegible for extended drug coverage plans. As a result of an inequitable and unjust healthcare system, ACB people rummaged and relied on a patchwork of social assistance programs and compassionate drug assistance to access HIV medication [23]. The processes of applying for these programs are time-consuming and bureaucratic, where eligibility depends on a physician providing evidence of an immigrant’s disability status. In some instances, ACB people involuntarily disclose their HIV status to social workers processing disability applications to increase eligibility chances. ACB immigrants who find themselves with expired IFHP coverages, who are ineligible for OHIP due to the three-month waiting period policy, or whose OHIP applications are pending fall into the health and drug coverage gap [32]. These legislative barriers result in ACB immigrants underutilizing healthcare and support services and delaying or interrupting ART. Studies show such practices could lead to increased viral replication, reduced chances of achieving viral suppression, poor health outcomes, and increased risk of HIV transmission [102].

Our study findings demonstrate systemic inequities including anti-Black racism, stigma and discrimination manifesting within social structures restrict the right to health for ACB communities. Unjust and discriminatory immigration, public health and healthcare legislative frameworks, policies, and institutional practices undermine and deny ACB people living with HIV the opportunity to access medically necessary services and support, obstructing human rights commitments, impacting health outcomes and quality of life, and hindering HIV response efforts. These injustices that produce and perpetuate structural violence for ACB communities are deserving of urgent attention and consideration by researchers, health and legal practitioners, and policymakers both nationally and globally.

Ratified international and regional instruments, legislation, and health profession regulations, guidelines, and protocols impose specific duties on states and all healthcare professionals to ensure the right to health and inform patients about their rights and options. All healthcare providers have a duty of care to their patients and a moral obligation to adhere to a standard of reasonable care when engaging in medical activities that can cause any form of harm to others. Physicians must comply with the Ontario Human Rights Code, RSO 1990, c. H.19 (the code), Regulated Health Professions Act, 1991 SO 1991 and the College of Physicians professionals’ standards when making any decision relating to the provision of health services. The Code articulates the right of every Ontario resident to receive equal treatment to services, goods, and facilities including health services without discrimination.

Our study findings advance legislative and policy response to addressing inequities and forms of structural violence constraining access to HIV healthcare and treatment among ACB people, and global efforts to end HIV “leaving no one behind” [6, 103]. Fundamental principles of universality and comprehensiveness that are the basis of the Canada Health Act and human rights must inform the provision of equitable healthcare in Canada. To address current legislative and institutional injustices and barriers in the HIV healthcare cascade and prevent onward transmission, Canada must ensure comprehensive and universal access to healthcare facilities and services. Implementing a national pharmacare plan would help address inequities and disparities in drug coverage in the province and different jurisdictions [30]. Healthcare providers should ensure that individuals have access to adequate information about HIV, understand the potential benefits of HIV testing and accessing timely healthcare, and provide informed consent before participating in the medical practices. The primary aim of HIV testing should be to promote and facilitate access to timely health and preventative care. This duty is owed to all persons without discrimination.

The barriers revealed in this study also highlight the need to develop an integrated system that ensures access to comprehensive and universal healthcare services [86]. The integrated system should consist of health system navigation models that would assist with bridging disjointed immigration, public health, health, and social support systems [104, 105]. Integrated models of care that include IME panel physicians, HIV specialists, general medical practitioners, public health, social support, social workers, community based ASOs, and patient navigators are relevant for improving linkage to care, streamlining patients flow within the system, and maximizing the delivery of HIV healthcare services. For example, the Client-Oriented New Patient Navigation to Encourage Connection to Treatment (project CONNECT) and the California Bridge Project strengthen linkage to HIV healthcare services [106, 107]. An integrated HIV care system will help to eliminate barriers associated with a lack of accurate information about Canadian healthcare services to immigrants, finding HIV specialists and physicians, booking appointments, and reducing long wait times. To end HIV/AIDS in Canada and globally, it is also critical to develop an innovative and integrated research strategy that combines behavioural and biomedical with structural interventions that address the unique needs of different populations [108]

## Conclusion

This institutional ethnographic inquiry centred ACB people’s standpoint to illuminate how their experiences accessing HIV healthcare and treatment are shaped by ruling relations within the healthcare system and beyond. Study analysis revealed that the day-to-day lives of Black people living with HIV are regulated by immigration, public health and healthcare systems, legislation, and institutional practices that are fragmented and unjust. Legislative frameworks, policies, and institutional practices of regulating these systems interface to disadvantage Black people living with HIV, limiting their right to health. Black people face structural barriers to timely linkage and engagement in HIV healthcare. The barriers call to question Canada’s commitments to the right to health and UNAIDS 90-90-90 target to end the global AIDS epidemic [9]. For Canada to achieve the global targets and end HIV, it must address inequities that continue to disproportionate impact Black communities at risk of and living with HIV and meet its commitment to the right to health. The provincial and federal governments must ensure non-discriminatory, timely, quality, and equitable access to healthcare and health services including HIV healthcare and treatment. This study has provided several recommendations for organizing Canada’s healthcare system as well as HIV healthcare and treatment practices. These findings further demonstrate the need for countries with pieces of legislation that violate human rights to develop or reform their policies and remove systemic barriers to healthcare and treatment services for marginalized communities living with HIV. The policy and practice changes can optimize HIV healthcare and treatment for ACB immigrants living with HIV in Canada and transnationally.

## Supporting information

S1 Text African, Caribbean, and Black people living with HIV interview guide. (PDF)

S2 Text Health workers interview guide. (PDF)
