# Supplementary material for: Unmasking legislative constraints: An institutional ethnography of linkage and engagement in HIV healthcare for African, Caribbean, and Black people in Ontario, Canada
Source: PLOS Glob Public Health. 2022 Sep 21;2(9):e0000714. doi: 10.1371/journal.pgph.0000714 (PMC10021522; doi:10.1371/journal.pgph.0000714)
Supplement: S1 Text — (PDF) [file pgph.0000714.s001.pdf]

Interview Guide: ACB people living with HIV in Toronto

Script prior to interview:

I would like to thank you once again for willing to participate in the interview as part of my study. As I have mentioned to you before, my study seeks to understand how the concept of HIV viral load creates conditions that inform how HIV care is organized, coordinated and delivered. My study also seeks to understand tensions and division between the lived realities of ACB people living with HIV who are seeking, accessing and engaging in HIV care and how HIV care is currently organized, and determine the consequences for ACB people. This interview will last at most 2 hours. The purpose of this interview is to get a comprehensive and concrete overview of the work you do to access and utilize HIV care services to get to undetectable HIV viral load. What I would like to do is "walk" with you as you provide a concrete description of the how you got connected to HIV care and the range of health services, for example HIV treatment, as well as what kinds of care experiences you get along the way. I am also interested in knowing if there are any challenges you face and any support you get to deal with the challenges.

You completed a consent form/ ***provided verbal consent*** *indicating that I have your permission (or not) to audio record our conversation. Are you still ok with me recording (or not) our conversation today? ___Yes ___No*

If yes: Thank you! Please let me know if at any point you want me to turn off the recorder or keep something you said off
the record.

If no: Thank you for letting me know. I will only take notes of our conversation.
Before we begin the interview, do you have any questions? [Discuss questions]
If any questions (or other questions) arise at any point in this study, you can feel free to ask them at any time. I would be more than happy to answer your questions.

Topic for Discussion with ACB people living with HIV

1) **Explore participants experiences and perspectives on Linkage to HIV Care**: Can you please walk me through your experience being linked or connected to HIV care in Toronto?

   Probes:

   a) Are you currently connected to HIV care in a healthcare setting in Toronto? If not;
      - Why are you not connected to care?
      - What are some of the challenges and barriers that you are encountering as you seek HIV care?
      - Are these challenges related to health coverage/insurance, getting a HIV healthcare provider, getting a pharmacist?
      - Are there any supports you are getting from any community organization to get HIV treatment/medication?
      - Has the lack of linkage to HIV treatment and care impacted your health and overall wellbeing in anyway? How so?

   b) If you are connected to care
      - How did you get linked or connected to HIV care? Were you connected or referred and by whom? Were you referred to a healthcare provider or a specific clinic?
      - Are there certain conditions or requirements you had to meet before being connected to care? What are these conditions? E.g. you had to have health coverage/insurance, referrals etc.

- Were there any challenges or barriers you encountered while seeking HIV care? E.g. getting access to healthcare provider-HIV specialist, family doctors etc.?

2) **Explore experiences and perspectives on accessing HIV care**: Can you tell me about your experience with HIV care once you were/are connected to HIV care.

Probes:

a) What HIV care services are currently accessing?
   - Are these services grouped/packaged in a particular way or are they single services accessed each individually? E.g. consultations, counseling, blood work, medication, treatment of HIV-related issues.

b) How do you go about getting these services?
   (i) Scheduling of appointments?
      - Do you have to schedule appointments with the health provider? E.g. family doctor, HIV specialists, pharmacists, lab technician?
      - How do you go scheduling the appointments? E.g. Do you have to do it by yourself, by phone or face to face? If not, who schedules the appointments for you?
      - Are there issues or considerations that determine how often you schedule an appointment with a healthcare provider-primary physician/HIV specialist, how often you refill your medication? E.g. are these determined by your general health, viral load measures, treatment adherence etc.?
      - Are there times or situations you have faced challenges or troubles scheduling appointments with health care providers? What are these challenges?

   (ii) Once you schedule an appointment,

      - Who do you see or interact with? E.g. HIV care providers- specialists or physician providing HIV care, nurses, social worker, other staff workers, pharmacist
      - How do you see them? E.g. do you see only one person or a team of health providers?
      - When do you see the provider or team of healthcare providers and how often? E.g. day of the week, at what time intervals, once every month, every 3 months etc.?

   (iii) What kind of things are done to your body when you go for your appointment?
      - Are there certain tests, procedures, blood work, screening and medical examinations that you undergo? What are they?
      - Are these standard procedures that are done on every appointment or are they based on need or risks?
      - How do you go about getting these procedures or activities done? Do you have to do them all at the same time during the appointment or you must book separate appointments?

c) Are there situations or moments when you have felt uncomfortable or often feel uncomfortable or frustrated as you access HIV services or engage in HIV care? What makes these moments troubling for you?

d) Are there times you feel your "race" is used in any way as a label to identify or direct how you receive HIV care?

3) **Explore experiences and perspectives on ART/HIV Treatment**: Can you tell me how you go about getting HIV medication.

<u>Probes</u>

a) How do you get your prescriptions for HIV medication? E.g. do you get it from your healthcare provider and how often?
b) Once you get prescription from your doctor, how do you go about getting your medication?
   (i) Where do you take it and what happens next?
   (ii) How do you pay for your prescription? Do you pay for it out of pocket, or do you have an insurance or ODSP?
   (iii) How often do you have to refill your medication? E.g. monthly, every three months
c) Are there times when you have found it difficult obtaining prescriptions from providers? Please explain.

d) Are there any challenges, troubles, and barriers you encounter getting and/or paying for HIV treatment?

e) Are there resources, documents, materials or information about HIV treatment that you are provided with? E.g.
   - How to take your medication? E.g. Time, daily, with food
   - When to take your medication?
f) Do you find yourself sometimes not taking your medication as prescribed e.g. with food, not on time, or sometimes missing your medication?
   - How often do you miss your HIV care appointments and HIV medication?
   - What causes you to miss your daily medication or not take your medication as prescribed?
   - are the challenges or barriers you face that impact how your take your medication?
g) Are there any challenges related to medication that you find troubling? E.g. side effects, drug interactions.
   - Do these challenges impact how you access HIV care and take your medications? Please explain

4) During your HIV care, are there certain educational resources, documents, guidelines or texts you are provided with by healthcare providers e.g. resources on how to take care of yourself, how to take medication etc.

5) **Explore knowledge of and perspective on undetectable HIV viral load**. Can you please tell me how you understand the idea of HIV viral load?

<u>Probes</u>:

a) What does HIV viral load mean to you?
b) Is your HIV viral load undetectable or detectable?
c) If detectable,

i. Why do you think you are detectable?
ii. Are there challenges you impact your ability to reach undetectable viral load? What these challenges are you encountering?
iii. Are you receiving any type of support from your healthcare provider or the institution you are accessing HIV care from? What types of support?
iv. Are there other types of support or services you need? What are these services

d) If undetectable,
   i. How did you achieve undetectable viral load?
   ii. What do you do/did in effort to get to undetectable and sustain it?
   iii. Are you receiving any type of support from your healthcare provider or the institution you are accessing HIV care from? What support?
   iv. Are there other types of support or services you need? What are these services
   v. Are there challenges your facing in effort to remain undetectable? What these challenges are you encountering?

6) **Explore experiences and perspective on patient-provider relationship:** Please tell me how your relationship with healthcare is like.

   Probes:
   a) How is your relationship with your healthcare providers like e.g. relationship with your doctors, nurses, pharmacists and/or other healthcare staff?
   b) Are there certain situations or issues that have influenced how you relate with your health care providers? Please explains
   c) Is there certain information about your health and life that you feel uncomfortable sharing with your healthcare providers? Kindly explain? And why do you feel the discomfort?
   d) Are there certain situations or issues that have made you feel healthcare settings are not safe places to share sensitive information about your health and life? Why so?
   e) Do you feel how you relate with your healthcare providers influence how you experience HIV care? How so?

7) Explore participants' perspectives on social and structural barriers and pathways for ACB people living with HIV to access culturally safe and coherent resources that address HIV vulnerabilities, promote HIV access and attainment of undetectable viral load and optimal health.

Probes:
a) Are there specific health-related needs and priorities you require? What are they?
b) Are there other types of healthcare services that you need that not provided during routines HIV care? Kindly Explain
c) Do feel your HIV care environment provide you will all the services and resources you need for your overall HIV care and wellbeing? What are some of services and resources that you need but and not available?
d) Are there any other challenges, roadblock, tensions, or issues that you experience or encounter in the course of accessing and receiving HIV care and services?
e) Are there any other types of support you would like to receive from your healthcare providers to improve your experience with HIV care? Please explain.
