# Supplementary material for: Unmasking legislative constraints: An institutional ethnography of linkage and engagement in HIV healthcare for African, Caribbean, and Black people in Ontario, Canada
Source: PLOS Glob Public Health. 2022 Sep 21;2(9):e0000714. doi: 10.1371/journal.pgph.0000714 (PMC10021522; doi:10.1371/journal.pgph.0000714)
Supplement: S2 Text — (PDF) [file pgph.0000714.s002.pdf]

Interview Guide: Healthcare Providers, staff, and policy/decision makers

Script prior to interview:

I would like to thank you once again for being willing to participate in the interview as part of my study. As I have mentioned to you before, my study seeks to understand how the concept of HIV viral load creates conditions that inform how HIV care is organized, coordinated and delivered. My study also seeks to understand tensions and division between the lived realities of ACB people living with HIV who are seeking, accessing and engaging in HIV care and how HIV care is currently organized, and determine the consequences for ACB people. This interview will last at most 1 hour. The purpose of this interview is to get a comprehensive and concrete overview of how the concept of HIV viral load structure your work of providing HIV care to ACB people living with HIV and the work you do to provide HIV care. I am also interested in learning about any tensions and challenges you face as you provide HIV care to ACB people.

You completed a consent form indicating that I have your permission (or not) to audio record our conversation. Are you still ok with me recording (or not) our conversation today? ___Yes ___No

If yes: Thank you! Please let me know if at any point you want me to turn off the recorder or keep something you said off
the record.

If no: Thank you for letting me know. I will only take notes of our conversation.
Before we begin the interview, do you have any questions? [Discuss questions]
If any questions (or other questions) arise at any point in this study, you can feel free to ask them at any time. I would be more than happy to answer your questions.

Topic for Discussion with Healthcare Providers and Staff

1. **Explore perspectives on organization of HIV Care**: Can you tell me how you understand HIV care is organized in your institution?

   Probes: (

   a) How are ACB people living with HIV linked or connected to HIV care services e.g. who connects them and how, are they referred, do they call the clinic or walk in?
      (i) How do they get connected to you or your institution e.g. E.g. did someone or an institution refer them?
      (ii) Is it easy for ACB people to be connected you as a provider or the institution? How so?
      (iii) Are there certain conditions, requirements, guidelines, or situations that determine who you take as a patient or who is connected to care at your clinic? What are these conditions?
      (iv) Has these conditions, requirements or guidelines impacted how ACB people are linked and engaged in HIV care? How so?

   b) Once ACB people living with HIV are connected to HIV care, what types of services do you or your institution provide?
      (i) Are these services grouped in a particular way? Are they standard practices of HIV care or are they grouped based on need and risks?
      (ii) Are the services HIV specific, HIV-related or general healthcare? Please explain

   c) How are these services accessed or provided to patients?
      (i) Do they have to schedule appointments? If yes With whom?

(ii) Who do they see? E.g. do they see only one person or a team of health providers?
(iii) When do they see them and how often? E.g. day of the week, at what time intervals?
(iv) Are there challenges ACB people living with HIV face scheduling appointments? What are the challenges?
(v) Are there challenges or troubles ACB people living with HIV face scheduling appointments with healthcare providers? Please explain
(vi) Are there issues or considerations that influence how often a person is scheduled for a HIV service? E.g. the general health, viral load measures, etc.
(vii) Is “race” used in any wat as a label to identify or direct HIV care that ACB people receive?

d) Who is responsible for the various aspects of HIV care for ACB people living with HIV? I’m interested to know about the division of labor and how roles are differentiated amongst the team, and also how sometimes communication or coordination could break down E.g.
   (i) Who is responsible for ordering tests, reviewing test results, communication of results to the patient, connecting ACB people with relevant providers/specialists/referrals, etc.?
   (ii) Who is responsible for coordinating other aspects of healthcare, e.g. medications, treatment of other comorbidities?
e) Documenting care for ACB people living with HIV-
   (i) What do you do to keep track of their care, e.g. how do you document what care they’ve received, what test have been conducted, keeping track of patients’ appointment etc.?
   (ii) What are you responsible for as it related to documentation and monitoring?
   (iii) Are there documents, guidelines, or texts that organizes and coordinates how documentation is done?
f) Is there anything about how HIV care is organized that you think makes it easier or more difficult for ACB people to attend or keep up with HIV care appointments? E.g. accessing all HIV care services in one place, seeing different doctors, lab hours for blood work, location of pharmacies, monthly visits to pharmacy?
   (i) Are there situations that patients miss clinical appointments? What happens when this happens? Do you or your clinic follow-up with them?
   (ii) What role do you play in ensuring ACB people get and commits to the required appointment? E.g.
      a. Do you or your institution provide any support or liaise with other community organization to support patients who are facing challenging attending their appointments?

2. **Explore the concept of HIV Viral Load**: Can you kindly take me through how the concept of HIV-viral load shapes, organizes and structures your work?

<u>Probes</u>:

a) What does HIV viral load mean to you?
b) How does it structure your work?
c) Are there any challenges that impact ACB people’s ability to reach undetectable viral load? What these challenges are you encountering?

d) Is there any type of support from you as healthcare provider or your institution that ACB people are accessing to enhance their work of getting to undetectable? What types of support?

3. **Explore work experiences and perspectives on providing HIV care to ACB people living with HIV**: Can you also tell me about your daily experience of providing HIV care to ACB people living with HIV including refugee, protected persons, immigrants, international students?

   Probes:

   a) Do you or your institution have services and health care practices that are specific to ACB people living with HIV? Please explain
   b) Based on your experience, what other types of services do you think are needed to improve HIV care specifically for ACB people living with HIV?
      (i) What are you or your institution doing to ensure these services are available and accessible?
   c) How is your patient-doctor relationship like with ACB people living with HIV?
      - Are there situation or practices within HIV care that you feel influence or impact your patient-provider relationship e.g. what information they share and how they share information? What are they?
      - How has these impacted how you provide HIV care services? And what are the resulting consequences?
   d) Based on your experience of HIV care, what are some of the challenges, tensions and barriers that you and/or your healthcare institution have encountered in providing HIV care to ACB people living with HIV including their ability to attain undetectable viral load and optimum health?
   e) How do you go about assessing these challenges?
   f) How do these challenges experienced by ACB people impact their HIV care and overall health including attainment of undetectable viral load?
   g) Do you think there are needs specific to ACB people that would improve their HIV care access, engagement and retention? What are these needs?
   h) What strategies do you think are needed to enhance HIV access and efficacy of HIV treatment among ACB people living with HIV?
   i) What role as a HIV care provider do you play to ensure the challenges are addressed, needs met and these strategies implemented? And what challenges do you face as a physician in trying to address their challenges and meet their needs?

4. Are there clinical, professional or institutional texts, policies guidelines, regulations, and standards that coordinate, regulate or govern how HIV care is organized and provided in your institution and profession?
   (i) What areas of your HIV care work is influenced and impacted by these guidelines, regulations, policies and/or standards?
   (ii) How has these texts influenced your ability and capacity to provide quality HIV care to ACB people living with HIV?

5. Are there any other institutional challenges and/or barriers that you feel prevent ACB people living with HIV from being timely linked or engaged in HIV care?

6. Are there tools, techniques and strategies that facilitate and support you and your institution in providing enhanced HIV care to ACB people living with HIV?

7. **Education and Training**: I would like to know more about any education and training you've had related to Black health and HIV, and how you've been trained to care ACB people living with HIV.

Probe:

a) Can you tell me about what training you've completed to be in your current position as a HIV healthcare provider or specialist or policy/decision maker? When was it? Where was it? What courses did you complete? Was there a practicum / internship / residency?
b) Have you ever received any training or formal education about HIV and Black health? Or formal education related to HIV and Black health? E.g. training on factors increasing vulnerability to HIV risk and comorbidities for ACB people? Or treatment options of chronic diseases in relation to genetics and cultural factors? What was it?
c) If no formal training, how have you come to understand HIV as a staff/healthcare provider, and what sources have informed your understanding? (Media, courses, colleagues, personal experiences, etc.)?
d) How have you come to understand HIV treatment, undetectable HIV viral load and comorbidities in the context of HIV and in relation to Black health (either through formal education or other things you've been exposed to)?
e) How do you believe HIV and race is understood amongst your colleagues and team? Members in this healthcare setting? E.g. How is HIV talked about / referred to? E.g. is it understood as a health condition that is comparable or equivalent to other conditions or issues you see in your practice? Or a unique condition and experience? What terms / language are used to talk about HIV?
f) How are ACB people living with HIV viewed or perceived in the context of HIV among your colleagues, team or within your healthcare setting?
